# After 150 years of watching: is there a need for synthetic ethology?

**DOI:** 10.1007/s10071-022-01719-0

**Published:** 2022-11-29

**Authors:** Judit Abdai, Ádám Miklósi

**Affiliations:** 1 ELKH-ELTE Comparative Ethology Research Group, Budapest, Hungary; 2 Department of Ethology, Eötvös Loránd University, Budapest, Hungary

**Keywords:** Comparative cognition, Synthetic ethology, Problem solving, Animal cognition

## Abstract

The Darwinian idea of mental continuity is about 150 years old. Although nobody has strongly denied this evolutionary link, both conceptually and practically, relative slow advance has been made by ethology and comparative psychology to quantify mental evolution. Debates on the mechanistic interpretation of cognition often struggle with the same old issues (e.g., associationism vs cognitivism), and in general, experimental methods have made also relative slow progress since the introduction of the puzzle box. In this paper, we illustrate the prevailing issues using examples on ‘mental state attribution’ and ‘perspective taking” and argue that the situation could be improved by the introduction of novel methodological inventions and insights. We suggest that focusing on problem-solving skills and constructing artificial agents that aim to correspond and interact with biological ones, may help to understand the functioning of the mind. We urge the establishment of a novel approach, synthetic ethology, in which researchers take on a practical stance and construct artificial embodied minds relying of specific computational architectures the performance of which can be compared directly to biological agents.

## Introduction

Students of cognition face a very difficult task. They aim to understand the structure of mental/cognitive processes that are impossible to observe or measure objectively by means of a measurement tool. The main tool used by behavioural scientists has been their own mind. Spence et al. (2017) argue convincingly that students of the animal (and human) mind cannot forget that they are humans. Human psychological theories powerfully influence what researchers think/believe about animal minds. The human mind is intentionally or unintentionally used as a reference point for many mental process or abilities displayed by non-human animals. In addition, during the last 150 years, changes in our relationships with animals and changes in our thinking about our mind have also had a direct effect on how the animal mind is conceptualised today (see Cambridge declaration on consciousness 2012).

Our own subjective influence has captivated social cognition in particular where anthropocentric concepts still dominate. Although one could argue that over the years there has been an advance on the (experimental) methods used and the statistical analysis applied in data analysis, few fundamental innovations have been witnessed.

In practice, ethologists’ data originate from either observing animal behaviour in nature or from watching animals closely in the laboratory in carefully controlled and manipulated situations. To use a somewhat loose analogy, the situation is like finding out the structure of some software by systematically pushing the buttons of a computer keyboard. Behaviour or performance is not a direct measure of cognition, because they are influenced by a plethora of (known and unknown) intrinsic and environmental variables. Although these raw data are then subjected to sophisticated statistical analyses, the results usually undergo a complex interpretational process to be useful as evidence for (or for the lack of) some cognitive phenomena. However, as can be witnessed for 150 years, we continue to have the same debates again and again on the nature of cognitive processes and how these control behaviour.

The situation gets even more complicated when one wants to explore the comparative aspects of cognition (cf. ‘comparative cognition’, see e.g. Beran et al. 2014), because the general processes attributed to cognition, for example, perception, acquisition, or memory, are subject to very divergent input and output processes in different animals, including humans. Input in the narrow sense may refer to the various sensory mechanisms (having a specific evolutionary history, see sensory ecology: Gibson 2014). Similarly, the output, behaviour/performance is also subject to similar evolutionary constraints in terms of mechanistic processes (e.g., degrees of freedom for motor structures). Thus, it is also a viable hypothesis that despite neural homologies, cognitive algorithms may also diverge between species.

In summary, present-day research on animal cognition faces the following challenges:

1. Many functional cognitive concepts (e.g., perspective taking, theory of mind) may be applied to human minds, but their definitions seriously restrict comparative research because they cannot be quantified properly. This anthropocentric approach forces us to think dichotomically (“animals either have it or not”) and disregards the possibility of conceptually different evolutionary or ecological solutions. The notion of “rudimentary theory of mind” in dogs, indicates this problem well (Horowitz 2011). In this regard, we support Shettleworth’s (2010) suggestion to operationalize complex anthropomorphic concepts.
2. Similarities in behaviour or performance do not necessarily indicate that two (or more) species utilise the same cognitive mechanisms for solving the same problem. Such differences can be revealed only by careful experimental design or many further (“follow up”) investigations that are usually lacking after the demonstration of the phenomenon. For example, the similarity in performance of relying on the human pointing gesture in dogs and children is often overrated: in reality, dogs display a more limited cognitive capacity than 2–3 years old children (e.g., Lakatos et al. 2009).
3. The comparison of cognitive abilities of two or more species often violates basic rules of experimental design (e.g. Boesch 2007), and several independent variables are not (properly) controlled for or cannot be controlled for. For example, any significant difference emerging after comparing captive born adult chimpanzees (*Pan troglodytes*) and human children (Boesch 2007) does not always provide valid information considering that those two groups differ in age, experience, socialisation etc.
4. Comparative research in animal cognition aims to reveal evolutionary trends, but that is not possible by collecting data only in two or three species. MacLean et al.’s (2014) effort to compare 36 species in a study on self-control is impressive but it also raises problems of how subjects of various species should be compared when they may differ in experience, perceptual skills, motor skills etc.
5. Many of the investigated cognitive concepts and the methods of testing in non-human animals are based on problems that are mostly relevant from an adult human perspective. Thus, it is less surprising that animals underperform under such conditions, and their performance is often difficult to interpret, which leads to problems of explaining their behaviour in cognitive terms. Although one may argue that, for example, social systems share many common features, the comparison of social skills in chimpanzees and humans may lead to false conclusions because the major differences in their species-specific social life may affect the way of thinking, despite the fact of being enculturated to humans in captivity (see also Boesch 2007).

## Preference for hidden food in dogs based on human cuing

Although it would be difficult to provide a specific ‘meta-analysis' on any topic in animal cognition, let us take a specific example because many of our readers may not agree with our critical points listed above. The choice of the examples is more biased to the authors’ knowledge and experience rather than any suggestion that these are the most glaringly problematic cases.

Recently, two independent research groups (Catala et al. 2017; Maginnty and Grace 2014) have reported similar results on family dogs’ (*Canis familiaris*) performance in the ‘Knower-Guesser’ task (Povinelli et al. 1990). In this task, subjects have to make a choice based on human cuing (looking behaviour) when one of the humans has had the possibility of witnessing the hiding of food (‘Knower’), while the other has not (‘Guesser’). The ‘Knower-Guesser’ task was developed to study the possibility of mental state attribution (‘theory of mind’) in chimpanzees (Povinelli et al. 1990), and it has been the subject of quite extensive debate. The work by Catala et al. (2017), and Maginnty and Grace (2014) is significant because they established clearly the level of performance in family dogs. In all experiments reported by these studies, the dogs performed significantly above chance and chose the hidden food based on the cues provided by the Knower rather than the Guesser. Rather than going into the methodological details of the experiment, we focus on the way the performance (approx. 65–75% average choice preference depending on the specific experimental condition) was interpreted by the authors.

1. Although there is a quite general agreement among researchers that the ‘Knower-Guesser’ task is not an indicative test for mental state attribution (Heyes 1998), both papers use this cognitive skill as a point of reference for their interpretation. For example, “Altogether, the findings of the present study provide evidence that canines are able to react to what others can or cannot see” (Catala et al. 2017); “Arguably, the performance of dogs in the present study constitutes a functional ‘‘rudimentary theory of mind” (Maginnity and Grace 2014).
2. The above tendency is even more remarkable, because both studies state that “preference for the Knower in this critical test provides solid evidence for geometrical gaze following” (Catala et al. 2017) and “results add to evidence that dogs have a remarkable sensitivity to cues related to humans’ attentional state” (Maginnity and Grace 2014). The assumption that dogs are able to calculate humans’ line of sight is by itself a strong hypothesis based in these data, so there is no need for hypothesising even more complex and elusive mechanisms.
3. The replication of this paradigm in humans showed that this task can be solved based on (at least) three different mental mechanisms (Gagliardi et al. 1995). These insights came from the subjects’ verbal reports when they had to explain their preferences in the experiment. Accordingly, some people referred to mentalistic mechanisms (e.g., the other was seeing, knowing) or relied on behavioural cues (e.g., turning head, visible eyes). Indeed, there seem to be three different (not necessarily exclusive) cognitive strategies: (A) attribution of a mental state to the other; (B) calculating the other’s perspective; (C) relying on discriminative behavioural cues including their relationship with the context (e.g., both humans look in the same direction but one of them looks at an object while the other looks at the empty space). Since all versions of this test with dogs involved some elements that allowed the operation of the discriminative strategy, it is unclear how manifestation of the above mechanisms can be separated and any hypothesis of mental attribution is based on flimsy grounds.
4. It is also of little help when authors try to refer to mental mechanisms based on the group performance. For example, “dogs have a remarkable sensitivity to cues related to humans’ attentional state, which enables them to respond as if they had a functional theory of mind” (Maginnity and Grace 2014); “dogs would have had daily experience with watching their owners prepare food for them,… On such occasions, dogs would have had ample opportunities to view the ‘‘eye-object line’’ … between their owner’s gaze and the food (Maginnity and Grace 2014); “Although the lack of systematic changes … suggests that more than associative learning was involved, this possibility cannot be ruled out.” (Catala et al. 2017). Thus, while there is ample evidence for ‘simple mechanisms’, authors seem to be unhappy with such a conclusion. It should be emphasised at this point that any ‘simple mechanisms’ typically evoked by authors may turn out to be very complex if one thinks about in the form of computations needed.

In sum, both studies present important and convergent observations on dogs’ skill at relying on human behaviour in finding food in complex social situations. However, despite a relatively low, albeit statistically significant, performance in this test, both authors prefer a highly complex cognitive interpretation of the phenomenon. However, if humans (at an individual basis) also vary in their cognitive strategies, how can we be sure that dogs use mental attribution at all? The reference to “functional theory of mind” is also not helpful from a mechanistic point of view, because it does not imply any specific mental mechanism.

## Caching behaviour in ravens

Caching has an important function in the life of many bird species that have to live during periods of food scarcity. In social species, caching also provides a basis for inter-individual competition including the exploitation of others’ resources. Thus, it is of pivotal importance that caches remain concealed from others, and countermeasures can be taken if others may detect the hidden food (e.g., Dally et al. 2006).

Scrubjays *(Aphelocoma californica)* have been observed re-caching hidden food if their action has been observed by bystanders (Clayton et al. 2007). This very intriguing behaviour probably comes about as a result of some experience both with caching and being ‘robbed’ (see also Bugnyar 2013). The observations have led to some discussions regarding the underlying mental mechanisms (see also Barrett 2012, p. 31).

1. The original authors (Clayton et al. 2007) assume that this behaviour is an indication that scrubjays are able to take the perspective of the other bird, while Penn and Povinelli (2007) claim that the birds have learnt to re-cache if being watched. Note that both accounts rely on the same general experience of the birds, probably some form of associative learning that inevitably results in a mental state (or states) that controls the bird’s behaviour when it is in the relevant situation. This hypothetical mental state emerges as a result of the relevant experience and ensures rapid response. The only difference is that Clayton et al. (2007) refer to it as ‘perspective taking’, while Penn and Povinelli (2007) do not provide a label. It may be wrong to provide any label, because nobody can be sure about the actual ‘content’ of that state.
2. There are, however, problems on both sides of the argument. Clayton et al. (2007) may be too bold in attributing perspective taking, because it is not clear how this mental state could emerge after some uncontrolled experience, and whether it is in some sense equivalent to the perspective taking typically ascribed to adult humans. Penn and Povinelli (2007) could be also wrong because they provide no evidence that the experience gained about caching cannot lead to a mental state that stands for perspective taking. There is also no experimental evidence on the exact associative learning process that may eventually lead to the observed performance. The problem is that we do not know what kind of experience is sufficient or insufficient for an agent to develop perspective taking, and what the minimum criteria for showing perspective taking are. We only know that in children, this skill develops step by step over many years of extensive experience in the human environment (Flavell et al. 1981). Our knowledge is mainly descriptive, that is, we know that the performance develops but not what kind of input is needed and how it gets organised by the child’s mind over a scale of many years. But even if the actual mechanism was known, other animals may rely on different mental computation for achieving comparable performance.
3. Bugnyar et al. (2016) aimed to solve this deadlock by introducing a new experimental design. Compared to the situation when they were isolated from conspecifics, captive ravens displayed rapid caching behaviour both when they were observed by others visually, or when they could only hear conspecifics nearby who, however, had the chance to observe them through a small hole in the wall. Based on their findings the authors argued that even in the absence of direct visual experience, the ravens reacted to the fact that the others could potentially see them. Indeed, it is difficult to argue how experience of a small hole in the wall would make the ravens cache faster without assuming some knowledge (mental state) about the possible visual access of the others. However, results also revealed that when choosing the location of the caches, the ravens did not consider what could be seen if a conspecific peeps through the hole. This led the authors to argue that ravens have a “minimal” rather than a “full blown theory of mind” (quotation marks are from the original study). One may also argue that if the ravens are unable to estimate the viewing angle of the other, what is the use of a perspective taking skill beyond knowing that another raven maybe sees something? In addition, the field of vision of ravens, as in most birds, is about 300 degrees, so it seems to be much less important to compute the other’s perspective very precisely, because it can see almost “everything around” independently of its head orientation.
4. Even if one accepts that the ravens are able to compute the other’s perspective it is far from obvious what kind of input is needed for the emergence of such skill. Maybe an analogy can help here. Machine learning algorithms can be very efficient in solving specific problems, that is, for example categorising visual images. However, their skill of doing this depends on a few very well-known factors, with important consequences. The performance depends (among others) on (1) the number and range of examples used for the training, (2) the processes (mathematical models) implemented, (3) how the level of acceptance (“correct performance”) is set during the training, and (4) in what way the novel samples (to be categorised) differ from the original sample set. Importantly, one is usually not able to pinpoint the ‘state’ of the software that would tell on what basis the categorisation is done.

We do not argue that the mind functions like a machine learning system. This analogy was introduced only to show that even if one could control both the input-output (stimulus and behaviour) and one knows the software (cf. mental processes) it is very difficult to define appropriately the nature of the emerging state(s). Thus, it is probably too early to label a mental state as, for example, perspective taking (and there is also no pressure to do so), but similarly, it is a naïve assumption to reduce those states being the outcome of ‘simple’ mechanistic associative processes.

In summary, Bugnyar et al.’s (2016) observations are interesting because they reveal a new environmental cue (“the hole”) that may affect caching behaviour in ravens surrounded by conspecifics, but this finding is of relatively little help for construing a mental model. Perhaps repeating this experiment with other corvids and other caching animals, including mammals, could reveal how general this kind of indirect ‘audience’ effect is, and possible differences can be explained by ecological and social factors characteristic of the investigated species.

## An optimistic approach

The situations described above seem to reoccur regularly in ethology. One could argue that the history of research reveals a lot of progressive aspects, and we are on a good track, moving forward step by step. Heyes (2012) observes also that present issues could be cleared up more effectively if researchers acknowledged the following strategy:

1. There is a need for clearer hypotheses for both theories to be tested.
2. Experimental design should allow for pitting the two types of hypotheses against each other.
3. If one experiment is inconclusive, it should be followed up by a better improved design.

Although quite logical, we doubt that following this procedure solves the problem. Typically, it is rare that researchers of very different points of view sit down and aim to formulate the necessary (alternative) hypotheses. But even if they were open for collaboration the exact nature of the two perspectives prohibits a common ground. For the cognitive approach, it is very difficult to provide a testable hypothesis that excludes all alternative interpretations. For the associative account, it is problematic to find all those valid experimental conditions (‘controls’) that exclude the influence of other (cognitive) mechanisms while keeping the modelled learning process ‘simple’.

## Taking stock

The more than 100-year-old debates within ethology and comparative cognition have shown only a relatively slow progress in understanding mental processes and how mental evolution affected problem-solving skills in animals. It is very difficult to arrive at satisfactory quality of research when results are obtained on small samples, and diverse methods are being confounded by species-specific constraints which open the door for a wide range of interpretations. This deadlock can be solved only if novel approaches as well as novel methods provide different kind of data that introduce novel ways of conceptualising the mental aspects of problem-solving behaviour. Thus, the question is whether solely following our traditions will eventually bring us significantly further, or whether there is a need to introduce new approaches, to broaden the horizon even if they are in their infancy.

Importantly, any new idea may not immediately revolutionise a specific field. For example, Roy and Sherrington (1890) were the first who experimentally revealed that there is a link between brain function and blood flow. But it took 100 years to use this original insight to match local changes in brain activity to sensory input, perception and/or mental processing (Kwong et al. 1992). Because functional Magnetic Resonance Imagining (fMRI) represents a completely different method in neurobiology than what had been used before, the real impact of the original idea only manifested after the corresponding technological development had taken place. Thus, in our case, an open mind and much patience is needed.

We also have to realise that the range of agents with problem-solving skills is growing. There is a growing potential in robotics (e.g., biorobotics, social robotics) for developing skilful agents, and hybrid systems of biological and non-biological agents (cyborgs) may also emerge. While these efforts can be supported by insights in animal cognition, they will also provide a challenge of how to compare robotic and biological agents.

In line with the above issues, we suggest focusing on problem-solving behaviour, which at the same time offers a broader, more flexible comparative approach. Problem-solving behaviour reflects short and long-term changes (decisions) in behaviour when the animal (agent) is responding to regular states and conditions of the environment in a way that is on average advantageous for the individual. Thus, the study of comparative problem-solving investigates how performance in biological (and non-biological) agents has diverged during their evolution as a function of ecological challenges in support of fitness.

This approach has at least the following advantages:

1. The unit of comparison is behaviour/performance that can be measured directly by relatively objective tools, which have the potential to become automatised in the not-too-distant future.
2. It offers a common platform for all kinds of agents which aim to overcome certain problems they face in their environment.
3. It does not need to refer to ‘cognition’ which is an anthropomorphic term being used also as a synonym for terms like ‘thinking’.
4. There is no need to forcefully separate cognitive and non-cognitive or lower/simpler or higher/more complex mental processes.
5. The focus on problem-solving does not prohibit cognitive theories. But starting with ecological relevant problems, critically evaluated from an ethologically sound point of view, should canalise the otherwise very anthropocentric approach to cognitive interpretations.

In the following sections, we would like to show how this approach can lead to novel insights that overcome the old deadlock focussing on and contrasting animal and human cognition.

## The need for synthetic ethology

If ethology is the biological study of behaviour (Tinbergen 1963), there has to be a specific biological level of organisation at which behaviour can be explained (modelled). However, any model is only as good as the extent to which it represents the phenomenon, but in biology, this can be evaluated only if the model is put to test in the real world. The ultimate proof for the basic mechanism of DNA transcription came from studies in which all the components for the reaction were put together and the process was reconstructed in vitro (e.g. Noireaux et al. 2011). Understanding how biological systems work by building them from components, is the basic tenet of synthetic biology (e.g. Benner and Sismour 2005; Cameron et al. 2014).

There has been no shortage of modelling efforts in ethology and comparative cognition. Early models, especially in the case of ethology, were more behaviour-based (e.g., the hierarchical model; Tinbergen 1951), while some comparative psychologists focused more on the possible ways how stimuli and actions are represented in the mind (see above; Holland 1990). There has also been a trend to combine behavioural and mental/cognitive modelling (e.g. Toates 1998; Timberlake 2001). Importantly, these agendas were never intended for a test in the real world. Models emerging in ethology or comparative cognition (1) are usually too vague for being implemented, and (2) have not driven conceptually new approaches or (3) have not driven methodological innovations.

Importantly, there have been other attempts to provide mathematically grounded models that aimed to capture the complex relationship between input and output (based on experimental data) in mental functioning without referring to any concepts that are characteristic of cognitive processes. The most influential one was the Rescorla–Wagner model (Rescorla and Wagner 1972) of associative learning, aiming to predict how behaviour changes as a function of experience. The core of the model was a relatively simple equation but what made it truly attractive was its potential to also make some unexpected predictions. While many critics of this model were focusing on its failure to explain other instances of the stimulus–response relationship (e.g., extinction), it still provided an important early strategy to make computational mental models without the need to invoke complex cognitive constructs (e.g., memory) or to refer to any biological substrates (e.g., specific cells, their connections or areas of the brain). In parallel, model building also started in ethology (e.g., Metz 1974; McFarland and Houston 1981) but nowadays, these approaches are conspicuously absent from the cognitive ethological literature.

### Broadening the agenda of synthetic ethology

The original idea of ‘synthetic ethology’ emerged in publications by MacLennan (e.g. MacLennan 1992). Synthetic ethology was put forward to study animal behaviour by constructing 2D virtual synthetic organisms that are allowed to behave and evolve in a synthetic world. More specifically, MacLennan (1992) used the example of investigating the evolution of communication in computer-based animated systems. He argued that this simplified system, which is comparable to “real/natural” environments, offers a novel tool for investigating how signalling systems emerge. Although this approach can be advantageous for studying specific questions, simulation of 2D agents and their environment can only provide a very limited analogy for the highly complex 3D natural space, and important environmental factors and challenges cannot be included.

Our new agenda for synthetic ethology goes well beyond the approach suggested above. The mind comes with a specific body and thus mental processes should not be studied in isolation but as an integral part of the embodiment (Ziemke 2003; van Horik et al. 2012). Agents should not be simulated only in a computer but built from scratch in 3D with embodiment. Thus, these agents and/or the proposed systems can be tested under the same conditions as the modelled animal (Webb 2000), and only such artificial agents can be truly compared to their biological counterpart. We argue that synthetic ethology is not just one alternative method for understanding animal (and human) behaviour, but that building de novo embodied autonomous agents that are able to function in the real world and interact with both biological and artificial creatures, should be an essential method. Emerging mental models of problem-solving behaviour should provide the input for implementation in these synthetic agents, and the main aim of synthetic ethology is to teach us about the organisation of behaviour through learning by doing.

Turing (1950) proposed that if an observer is not able to distinguish the performance of a machine from that of a living being then, at least from the computational point of view, the two systems can be accepted as ‘equal’. This view has been criticised in different ways (Saygin et al 2000), especially because there is some possibility in the test for cheating. However, the Turing test can be also regarded as benchmarking test in engineering, when the performance of any two artificial systems/agents is tested and compared. Synthetic ethology also rests on this premise, that is, the comparison of performance in a specific situation, which is observed in the biological and artificial agents, provides the measure of similarity of mental power, irrespective of the actual mechanism.

In short, by revealing that a synthetic agent is able to solve the same problem as its biological counterpart, we could claim that we found at least one way of modelling its mind. Importantly, at the beginning of this kind of research we cannot be sure that the newly developed artificial mind and the biological mind are controlled by similar rules, but as more and more possible solutions are discovered (in relation to diverse problems), the more likely is that some mental algorithms show a closer similarity (Krakauer et al. 2017). Importantly, this test could not only be executed by human experimenters (from a 3rd person perspective), but also by the living organisms that we wish to model (2nd person perspective). For the sake of the argument, if an artificial agent is as successful in a colony as a typical bee, then we have at least one model for ‘beeness’ or “functioning like a bee”. We should keep in mind that such in silico models are in many ways fundamentally different from their biological counterparts. Thus, the aim is not to provide a “copy” of the biological mechanisms that are executed by the neural structure, but rather to provide a computational solution to the problem(s). Importantly, in contrast to earlier, purely descriptive notions of functional equivalence claimed by observing similar performance (see above), synthetic ethology would provide a genuine working mechanism that drives the artificial agent.

These computational mental models have at least two more advantages. First, they offer a common medium for comparing the problem-solving ability of various species and problems. Although there have been huge efforts to map brains in smaller or larger organisms (Rybak et al. 2010) and then build a reconstruction using computers, it will take some time until we have substantial information about all neurons, their connections and all possible types of interactions. Bees, for example, have about 1 million neurons (Rybak et al. 2010). Thus, an alternative approach is to take a more general hardware structure and aim to develop algorithms that are able to solve the respective problems.

Second, although it is not a simple task, such artificial systems, specifically their software also offer the possibility of being measured in terms of complexity (Fenton and Neil 1999). One may hypothesise that the complexity of the software would correlate with the complexity of the task to be solved by the biological organism. If the artificial agent is able to solve the same problem in a comparable way to the biological organism, then the parameters of the program can be used as an objective measurement for estimating the complexity of the problem (see also Cooper and Peebles (2015) for similar line of arguments). This could be important, because at present, we do not have objective criteria for judging task complexity in animal behaviour.

## Four foundations of novel approaches provided by synthetic ethology

Just as in the case of other similar approaches in biology, synthetic ethology is only in its infancy. Not only are the vision and concepts missing, but there are also significant practical limitations (see below). However, it is still important to provide a short summary about the novel possibilities that may play a crucial role in the emergence of synthetic ethology.

### Behaviour data en masse

Laboratory investigations usually involve 10–20 individuals (per group), and researchers typically measure a few specific behavioural variables and/or artificial parameters (e.g., going left/right, pushing a lever) when the focal animal is interacting with its environment (or some equipment) or with other companions. The method of data collection is tightly connected to available human resources, human skills (e.g., vision or hearing), and researchers aim to make experiments more reliable and reproducible by using inter-observer agreement as a tool for validation. However, it seems impossible to understand the mental mechanisms if only a few very specific input/output parameters are measured.

In the last 10 years, several novel methods have emerged that extend the possibility of collecting large amount of behavioural data. Sensors measuring activity of the animal are becoming widely available for behavioural research (Valletta et al. 2017). These sensors can be placed on the animal or in the environment. Sensor-technology offers the extension of present research methods by many magnitudes. Here, we mention only a few new features:

1. Large number of animals equipped with sensor can be ‘observed’ in their natural habitat for extended periods. Video cameras mounted on nest boxes, or movement sensors on wild or domesticated animals offer the possibility not just of collecting more data, but also of providing a view of hitherto unknown aspects of their life (for review see Wilmers et al. 2015).
2. The natural habitat can be modelled inside a laboratory with increased control, leading to better validity (e.g., IntelliCage; Iman et al. 2021). Although this method also has important limitations, in the case of laboratory animals, it avoids the typical problem of collecting data only from a single short test session and the effect of transfer from the home cage to the testing site (Iman et al. 2021).
3. The limited human resource currently used for direct observation can be deployed to analyse massive amounts of behavioural data.
4. Behavioural ‘big data’ offer the possibility of analysis in parallel at different levels, such as individuals, group of individuals or population/species.
5. Big data potentially enable us to recognise patterns that could not be detected in small, fragmented data sets.
6. Using the same technology reduces observer and laboratory biases, partly influenced by theories about the behaviour.
7. Direct comparison is possible between biological agents and their non-biological counterparts.
8. The analysis of these data could provide parameters that can be used in algorithms aimed at modelling mental processes.

Recent research has made huge advances not only in collecting such “big data” but also in developing the tools for analyses. Machine learning provides a novel opportunity for replacing human observers and extending the possibilities for extracting novel kind of additional information from sensor-based data (Anderson and Perona 2014). For example, using DeepLabCut (Mathis et al. 2018; Nath et al. 2019), a markerless pose estimation software, allowed researchers to investigate imitative similarity and spatial variability in the actions of an infant and an adult, thus providing unique information about the nature of infants’ problem-solving approaches (Solby et al. 2021).

New machine learning methods also allow us to recognise novel behaviour patterns not detected by human observers, and continuous recoding of behaviour patterns also provides the possibility of looking for neural correlates (e.g. Gomez-Marin et al. 2014). Successful applications of this methodology have established a field called computational ethology (Anderson and Perona 2014). Large amounts of behavioural data support post hoc generation of behavioural models that could be tested by in silico models of synthetic ethology.

This means that we can now go beyond reporting specific actions or choice behaviour (e.g., going left or right), and behaviour data can be collected ‘on the fly” in parallel with changes in the environment. Although this makes any analysis more complex, a richer account of the events may provide a much better basis to modelling attempts.

### The proof of the pudding is the eating

Computers are slowly reaching the computing capacity of the human brain. Thus, the problem of how much it can do is changing to how it does it, that is, models of the mind should provide algorithms that are able to solve the same problems faced by the human or non-human animal.

As a thought experiment, let us assume that we have an artificial scrubjay robot with perfect vision and object manipulating skills (to avoid the robot failing because it has visual or motor problems). Referring to the above example, for simplicity, one could run an algorithm based on the ‘simple association’ model: re-cache if another bird has observed the caching. Putting this robotic scrubjay among group mates, it would rapidly turn out how this computational algorithm fares under different situations, and whether its performance differs from that of the real birds if tested under artificial conditions (see above Bugnyar et al. 2016).

After collecting detailed data on raven caching behaviour, one might find that ravens achieve this performance after much less experience than the robotic raven. In terms of performance, this would mean that the biological raven is still better than the artificial one, because it needs less time/input/practice to achieve the same or better performance. This hypothetical outcome should, however, lead to important insights and to further experimental work to improve the artificial agent. For example, we could conclude that simple ‘en masse’ experience of a ‘simple association’ does not suffice (note that exactly the opposite is often used as an argument to dismiss cognitive accounts) and one has to find either a better algorithm or some other collateral mechanism that enhances the performance of the artificial agent. For example, it may be considered that there is a specific (perhaps in-build) mental tool that is able to recognise very specifically the head orientation of the other birds standing nearby (c.f. the face recognition software present on many mobile phones is doing something similar) and thus this could allow for a more specific context for (faster) learning.

When a logically similar experiment was carried out with a virtual agent simulating attachment between a dog and its owner in 2D (Vincze et al. 2021), optimal functioning that corresponded to the natural situation required the introduction of a lot more novel computational rules than assumed at the start of the investigation.

As this example shows, the critical aspect of such proof of concept is not the set of computational rules that are needed for controlling the behaviour but that we develop and build a mechanistically functional embodied agent. Bio-inspired approaches to build a “rat” (Wiles et al. 2012), “cricket” (Webb 1995) or “bee” (Michelsen et al. 1989; Landgraf et al. 2008) (see below) may offer a starting point for such experiments. Importantly, for many situations morphological similarity is not necessary. We have successfully used car-like robots for initiating social interactions with companion dogs (e.g. Abdai et al. 2015, 2017; Gergely et al. 2015).

An alternative method would be to directly feed the scrubjay robot with data obtained from thousands of re-caching events (and complemented with data from the actual social environment) evaluated by sensor systems that are deployed to detect this behaviour in freely interacting birds and see whether it is able to develop a satisfactory solution to the problem. This would also allow us to measure and compare the success of the biological and non-biological agent. A similar approach was used to develop a robot bee by Landgraf and colleagues (2008, 2010, 2018). They analysed the dance trajectories of real bees from hundreds of videos, using an automatic tracking system which provided information about the motion parameters and behavioural properties, facilitating the design of the hardware and motion of a robot bee (Landgraf et al. 2008). In their field study, the robot bee increased foraging motivation and could elicit following behaviour in honeybees; however, the authors indicated that the robot did not attract many followers (Landgraf et al. 2012, 2018). Thus, they found a difference in success between the biological and non-biological agents.

### In silico* evolution of the mind*

Evolutionary robotics offers an important way to get insight in the emergence of some cognitive phenomena (Floreano and Keller 2010). Among others, Floreano and his colleagues have developed a range of tools that can be used to find out what kind of skills evolve under specific challenging conditions. Investigating the evolution of simple communication systems under the influence of different factors (relatedness and signalling capacity), they found that deceptive strategies could also emerge (Floreano et al. 2007). While the authors implemented this experiment to find out about evolutionary scenarios that may facilitate different communication strategies, it seems that functionally deceptive behaviour may emerge also at low levels of complexity. This means that not deception per se but its ‘complexity’ or ‘context-independence’ should be the basis for any comparison (Shim and Arkin 2012). In line with this, it has been argued that although both corvids and apes can use sticks as tools, apes can use a much wider range of objects in very different contexts (Seed et al. 2009), and a difference of similar nature is also argued for when comparing non-human apes and humans.

Evolutionary robotics can also offer a substrate for competition between different algorithms of mental control (Floreano et al. 2008). If these algorithms controlled complex embodied systems (mimicking a biological agent), one would be in the position to find out which one is the best mental model for that particular function. Importantly, the evolution of the mind is a long-term optimisation process, because any species has to solve a set of different problems during its life. Thus, just as in the case of other evolved traits, there is a trade-off between highly specific performance in one task and sufficient performance in a wide range of situations. This provides us with an interesting scenario in which one could reveal the best mental model for a specific problem that does not interfere with performance in other situations.

### Real ‘immersing’ agents—the Trojan horse method

The field of animal–robot interaction should be regarded as a forerunner of synthetic ethology, existing studies using robots to investigate the problem-solving skills of animals in specific situations. This approach could be regarded as the continuation of the footsteps of Konrad Lorenz (1981) and Niko Tinbergen (1948) who used simple dummies and decoys. Artificial agents allow us to go a few, significant steps further by allowing the introduction of complex stimuli as well as deploying computational algorithms that control the behaviour of the artificial agents and make the experimenter’s intervention unnecessary.

**Fig. 1:**
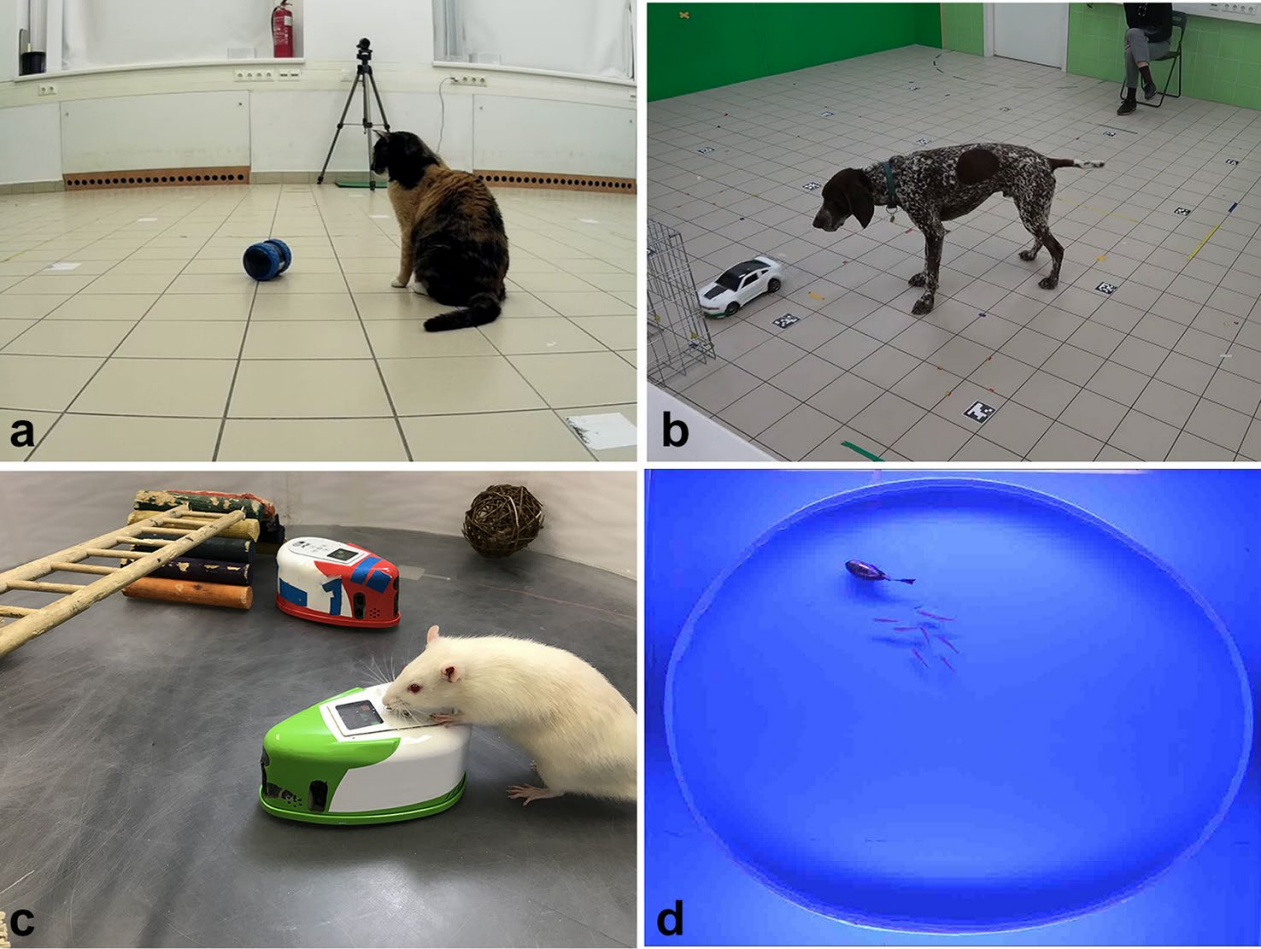
**A** A cat is approaching a UMO (Sphero Ollie) that previously displayed animate motion cues (Photo by Judit Abdai; Abdai et al. 2022b); **B** a dog is waiting for the UMO to retrieve food for him (Photo by Judit Abdai; Abdai et al. 2022a) **C** a rat is interaction with a robotic iRat (Photo by Laleh Quinn; Quinn et al. 2018); **D** a small fish shoal displays cohesive tracking toward the robot fish (Photo by Maurizio Porfiri; Aurelli et al. 2012)

Some of the robots have been used to test the behaviour of a single individual (e.g. Abdai et al. 2022a, b; Quinn et al. 2018); or they may be integrated into a group (e.g. Halloy et al. 2007; Landgraf et al. 2018; Jolles et al. 2020). Note that many of these situations can be envisaged as a specific Turing test mentioned above. If the individuals interact in the same way with the robot and their biological counterpart in the absence of any direct human influence, then the agent’s functioning represents one possible computational solution for the interaction (see Fig. 1).

In the area of animal–robot interaction, the most extensive literature belongs to the development and use of biologically inspired robot fish, probably because typically fish do not have complex movable extremities apart from the fins. Using such robotic agents, researchers have identified several important features that contribute to its acceptance as a social partner. For example, realistic eyes and natural swimming behaviour in guppies (*Poecilia reticulata*) (Landgraf et al. 2016), resemblance to the body shape (aspect ratio) of a zebrafish in zebrafish (*Danio rerio*) (Abaid et al. 2012) or mimicking the natural colour pattern and tail-beat frequency in golden shiners (*Notemigonus crysoleucas*) (Polverino et al. 2013) all improved the robots’ acceptance by conspecifics.

The Robofish have been used successfully to investigate specific aspects of the behaviour of different fish species in experimental situations which are very difficult to arrange among living individuals. Examples include whether larger leaders are more likely to be followed irrespective of the follower’s body size and risk-taking behaviour (Bierbach et al. 2020); how individual speed influences the collective behaviour without the confounding influence of potential socially induced changes due to interactions (Jolles et al. 2020); and the role of visual and non-visual cues on the social behaviours of two populations (surface- vs cave-dwelling) of the Atlantic molly (*Poecilia mexicana*) (Bierbach et al. 2018).

One of the most important aspects of animal–robot interaction research is the real-life application of the robot. In recent studies, Polverino and colleagues (2019, 2022) used the Robofish to test whether it can be used effectively against the mosquitofish, which is a highly invasive species. They found that a robot fish mimicking the behaviour of their native predators could elicit long-term negative effect on the survival, reproduction and ecological success of mosquitofish while having no influence on tadpoles (a species negatively impacted by mosquitofish in the wild) (Polverino et al. 2022).

In most studies using artificial agents as social partners, the robots have mimicked the studied species (or its predator) (see above). However, depending on the specific question studied, it can be advantageous to apply a robot whose embodiment does not resemble a conspecific (e.g. Abdai et al. 2018). In a series of studies, we have investigated the interaction in various situations between dogs and an unfamiliar, (seemingly) self-propelled object, referred to as a UMO (unidentified moving object). We used these robots to study the influence on the behaviour of dogs of specific behaviour cues in themselves, without the potential effect of previous experiences with the interactive partner (based solely on its dog- or human-like appearance).

Our dog–UMO interaction studies revealed that self-propelledness, using multiple trajectories, reactivity to dogs’ behaviour, and helping behaviour in a problem-solving task contribute to the acceptance of a robot as a social partner (e.g. Gergely et al. 2013, 2015; Abdai et al. 2015, 2017). Dogs displayed social behaviour toward UMOs when they encountered a problem-solving task, and following this short positive interaction, they rapidly learnt to follow the communicative indication of the UMO in a two-way choice task (Gergely et al. 2015), and the UMO was able to elicit social bias in dogs (Gergely et al. 2016; Abdai et al. 2022a, b). Importantly, dogs remembered the behaviour of the UMO even a month after the initial interaction (Abdai et al. 2022a, b).

These examples of the animal–robot interaction studies demonstrate that the deployment of simple agents can lead to important insights. Artificial agents integrated into one-on-one interactions or groups provide unique opportunities in research:

1. By transferring the behaviour of real animals into a robot based on mass data, we can test what is the nature of the algorithm that makes the agent’s behaviour the most similar to its biological counterpart.
2. Applying open-loop system (e.g. Faria et al. 2010; Polverino et al. 2012), in which the behaviour of the robot is pre-programmed, can ensure high control over the situation. It allows testing specific hypotheses by having behaviour programs that are congruent or incongruent with the behaviour expected in the specific context.
3. Closed-loop systems, that is, when the robot can react to the behaviour of the subject in real time (e.g. Kopman et al. 2013; Spinello et al. 2019) offer the possibility to find out how interaction with biological agents changes the parameters of the artificial mental model, that is, the role of experience can be quantified.
4. An intriguing new approach is the real-time transfer of a set of actions of an individual onto a remotely located robot (Karakaya et al. 2020) which facilitates the real-time manipulation of different factors in social interactions. This method allows us to manipulate the embodiment and behaviour separately, while also providing the robot with a more natural behavioural response than is possible using a mathematical representation of social behaviour (Karakaya et al. 2020). Recently, Karakaya et al. (2020) successfully established an interaction between two fish by the means of robots. In this experiment, they deployed two remote tanks, and in each, one live fish interacted with a robotic fish that exhibited the behaviour of the real fish in the other tank.
5. By building the robots and improving their mental algorithms step by step, we can measure what are the important physical or behavioural features that contribute to establish and maintain interaction with another agent, that is, for example, the duration and the intensity of the social interaction can be used as primary parameters for evaluating the artificial agent’s social skills.
6. Application of these agents allows researchers to study those details of an interaction that would be impossible when using real animals. For example, one can study how individual speed influences the collective behaviour without the confounding influence of potential socially induced changes due to the interactions (Jolles et al. 2020).
7. Application of robots as interactive partners may contribute to animal welfare by reducing the number of animals needed for research. For example, when investigating shoaling behaviour of a specific individual, there is no need to house other individuals used solely as partners. This method may be especially important in situations where the social interaction is potentially harmful to (at least) one of the participants, for example, when investigating aggression or predation.

## Limitations of synthetic ethology

Although building a biological agent “from scratch” would be the best option to compare it to its natural counterpart, it is unlikely to happen soon because even the making of an artificial chromosome is very challenging (Moralli and Monaco 2015). In addition, successful attempts would also raise serious ethical issues. Synthetic ethology has an advantage over synthetic biology, because we can utilise artificial in silico agents as modelling substrates.

We propose that synthetic ethology can provide a strong theoretical and practical basis for understanding animal behaviour, including mental processes, but presently, there are significant limitations. Although embodied agents are developing rapidly and there are commercially available autonomous robots with fairly sophisticated abilities (e.g., Boston Dynamics Spot®), there is still need for improvement in technology that establishes the complexity and skilfulness required to build de novo agents capable of functioning in real situations. Such limitations can be virtually overcome by ‘cheating’ in some cases, that is, certain aspects of the behaviour (reaction to the environment) are not modelled based on how a living entity functions, but rather designed with respect to the current state of robotics (e.g., using wheels instead of legs when the means of ambulation is not the focus of the study). But depending on the specific aims, this may not be as much of an issue as it seems:

1. These limitations may help in finding the minimal skill set required for the artificial agent to function and survive. For example, when the aim is to build an agent that can function and survive in a specific new niche, and thus it has no natural counterpart in the form of a biological organism, these constraints may even help us avoid designing overly complicated systems.
2. Considering that we do not have detailed information about the underlying cognitive mechanisms of many behavioural functions, having constraints in building novel agents may provide novel perspectives.
3. Having known limitations and knowing where we “cheated” and to what extent, provides still more information, compared to what we know about animals just by observing their behaviour.
4. The substantial difference in the substrate (organic vs inorganic compounds) of biological and artificial agents makes any close resemblance at all structural levels impossible: as far one can see now, computers cannot provide a direct copy of natural minds. Thus, we do not assume that any artificial agent provides a close similarity to their biological counterpart. Nevertheless, the application of such agents can offer a tool that helps us distance ourselves from our own mind.

## Conclusions and future directions

Some readers may doubt that our scenario is realistic. However, there are clear indications that all kinds of embodied agents can be constructed, from relatively simple agents like cars and mobile phones to more complex ones, for example, Mars rovers. Specifically, the last of these should behave in many ways like animals to be successful on a novel terrain. Problem-solving skills in animals, modelled by artificial agents, could be very helpful for exploring a new planet. In addition, the emergence of various novel approaches like computational ethology and evolutionary robotics can help a lot in moving us from a qualitative inquiry (human observation of animals/agents) to massive quantitative research combined with insights from neuroscience and mathematics. Thus, ethology could leave behind the image of being a science of the early twentieth century and join the biological revolution we are all witnessing today.

A major challenge and limitation of synthetic ethology is the human element, that is, researchers interested in the behaviour of animals are not keen to learn about robotics, and roboticists like to construct and program artificial agents rather than studying animal behaviour. Thus, the main goal of synthetic ethology could be to bring together these two different areas of expertise and initiate new avenues of joint research. The authors believe that the future (or the beginning of synthetic ethology) is closer than many of us think.

Our idea needs improvements in technology if it is to fulfil its expected promise. Therefore, beyond introducing the promises of this approach, we also urge behavioural scientists to ‘get involved’ in this line of research, especially because its interdisciplinary nature requires us to ‘step out of the box’ and become more familiar with computer technology, programming, machine learning and the like. This novel challenge provided by synthetic ethology could be attractive for young students who could be disillusioned by the agenda of current comparative sciences of the mind (Abramson 2015). The epoch of synthetic ethology will begin if ethologists and comparative psychologists will watch their favourite animal species with the explicit goal of using this knowledge for developing and constructing minds of artificial agents.
